# Development and Practice of Sports-Related Public Welfare Platform Based on Multi-Sensor Technology

**DOI:** 10.3390/s23020713

**Published:** 2023-01-08

**Authors:** Quantao He, Tongchang Hu, Yong Zhong, Wenjuan Li, Ren Sun

**Affiliations:** 1 Sport School, Shenzhen University, Shenzhen 518000, China; 2 Football School, Wuhan Sports University, Wuhan 430079, China; 3 School of Sports Leisure, Beijing Normal University at Zhuhai, Zhuhai 519000, China; 4 Faculty of Physical Education, Beijing Institute of Technology at Zhuhai, Zhuhai 519000, China

**Keywords:** sports public welfare platform, multi-sensor technology, Kalman filtering, multi-sensor data fusion

## Abstract

Today, more and more Internet public media platforms allowing people to make donations or seek help are being founded in China. However, there are few specialized sports-related public welfare platforms. In this paper, a sports-related public welfare platform that aims to help people who were disabled due to participation in sports and those who are disabled but want to participate in sports was developed based on multi-sensor technology. A multi-sensor data fusion algorithm was developed, and its estimation performance was verified by comparing it with the existing Kalman consistent filtering algorithm in terms of average estimation and average consistency errors. Experimental results prove that the speed of the data collection and analysis of the sports-related public welfare platform using the algorithm established in this paper was greatly improved. Relevant data on how users used this platform showed that various factors affected users’ practical satisfaction with sports-related public welfare media platforms. It is suggested that a sports-related public welfare media platform should pay attention to the aid effect, and specific efforts should be devoted to improving the reliability and timeliness of public welfare aid information, and ensuring the stability of the platform system.

## 1. Introduction

In recent years, scandals involving the Red Cross Society of China, such as misappropriation and embezzlement of donations, have decreased people’s trust in traditional official public welfare organizations. People are more willing to use online public welfare media platforms that offer open information about the progress of each fundraising project, the use of donations and donation feedback, and they also feel more at ease and more engaged with them.

Relevant studies have been conducted on online public welfare media platforms. Zhang C analyzed and predicted the forwarding scale of users of the Sina micro-public-welfare platform by establishing an integrated T-S system prediction model that integrated a BP neural network algorithm, an RBF neural network, and a neural network [1]. The simulation results show that this model has a better generalization ability and improved prediction accuracy compared to the BP and RBF networks. Pallesen E explored how sharing economy was used as a path to welfare innovation based on a case of activating a digital platform to support sharing among citizens with lung disease to improve their health and well-being [2]. The empirical case exemplifies that public actors are currently embracing the sharing economy in an attempt to extend the goals of the public sector beyond itself, and attention should be paid to how sharing interacts with technology in unforeseen ways and with unexpected outcomes. Shen et al. investigated the influence of Internet commonweal characteristics on consumers’ participation willingness using the project Ant Forest issued by Alipay, as an example [3]. The findings show that openness and interactivity, two characteristics of Internet commonweal, have a positive impact on customers’ co-creation value, and co-creation can work as a mediator of the relationship between Internet commonweal characteristics and customers’ participation willingness.

These studies focused on the relationship between online public welfare media platforms and their users. However, there is an insufficiency of studies on how artificial intelligence, especially multi-sensor technology, can be applied to improve online public welfare media platforms and promote user satisfaction with platforms. Additionally, there is a lack of studies on sports-related public welfare platforms. In this study, multi-sensor technology was introduced and used to develop a sports-related public welfare platform that aims to help people who were disabled due to participation in sports and those who are disabled but want to participate in sports. A multi-sensor data fusion algorithm was developed, and its estimation performance was verified by comparing it with the existing Kalman consistent filtering algorithm in terms of average estimation error and average consistency error. Relevant data on how users used this platform were collected and analyzed to determine the factors affecting users’ practical satisfaction with public welfare media platforms.

## 2. Multi-Sensor Technology and Related Works

Multi-sensor technology uses computer technology to automatically analyze and synthesize information and data from multiple sensors or multiple sources under certain criteria to complete required decisions and estimates. 

Da et al. outlined recent advances in multi-sensor multi-target tracking based on the random finite set (RFS) method and emphasized relevant research topics and some remaining problems [4]. In multi-sensor filtering, data-level multi-target detection fusion and estimation-level multi-target density fusion both play an essential role and ensure the sharing and fusion of local measurements and posterior densities between different sensors. The optimality and sub-optimality of each fusion rule and different RFS algorithms were presented in detail. Lie et al. studied the impact of heterogeneous technology integration on 3D integrated image sensors [5]. Their study discussed a system-level model that analyzed the correlation between design choices, power, performance, thermal, and noise. A 3D image sensor was analyzed as a case study, and light sensor modules designed in a 180-nanometer process were considered. Coupling analysis showed that scaling down the compression engine to a deep submicron process provided higher throughput for a given system power or lower power for the entire target. Chip-to-chip thermal coupling could be used to adjust the quality of compressed images. Laleicke P F and Kamke F A developed a four-channel planar capacitive sensor and calibrated it on a maple veneer [6]. The calibration material was conditioned to equilibrium at three different relative humidity levels. The optimal penetration depth was determined by statistics and analysis. A mixed-rule method and a volume model were applied to derive the moisture content of the different layers. Different degrees of fit were observed between and within the different electrode and calibration datasets. The sensor was able to determine changes in moisture content and positive moisture content gradients rather than averages, but it had limitations in detecting negative moisture gradients. Lu et al. investigated the optimal-state estimation problem for the fusion of asynchronous multi-stage multi-scale sensors with unreliable measurements and correlated noise [7]. Noise from different sensors was cross-correlated and coupled with system noise from the previous and the same time steps. The system was described with the highest sampling rate, and different sensors independently observed a target at multiple sampling rates. Their study proposed an optimal-state estimation algorithm based on white-noise estimator iterative estimation. The algorithm effectively utilized the observation information, overcame factors such as packet loss, data failure, and unreliability, and improved the accuracy and robustness of system state estimation. 

This study attempted to use multi-sensor technology to develop a specialized sports-related public welfare platform that aims to help people who were disabled due to participation in sports and those who are disabled but want to participate in sports and help improve user satisfaction with the platform. 

## 3. Development of Sports-Related Public Welfare Platform

Internet public welfare includes micro-public welfare, Internet + public welfare, and online public welfare. The Internet can be used as a platform for public welfare communication. Online public welfare platforms combine an online platform and traditional public welfare, and attention should be paid to research on evaluation standards for platform development [8]. Three main aspects are taken into consideration when developing such Internet platforms [9]. The first one is concerned with developing the platform to increase the value creation generated by the users. The second is focused on proposing new technical improvements for the platform’s development. The last one concentrates on how to attract users and improve user experience.

In China, there is a large population of disabled people. The disabled people’s federations at different levels are devoted to assisting disabled people, reflecting their needs and safeguarding their rights and interests. However, there is no specialized sports-related public welfare platform using multi-sensor technology to help people who were disabled due to participation in sports and those who are disabled but want to participate in sports.

To develop such a platform, the disability assistance resource pool should first be clarified. The disability assistance resource pool includes disability assistance funds from the China Disabled Persons’ Federation, corporate sponsorship funds, and various materials. Additionally, it is necessary to aggregate the forces of the China Disabled Persons’ Federation, enterprises, and volunteers to form a service chain to provide a direct on-demand service supply for the disabled. In the process of serving disabled people and promoting the progress of social assistance, institutions and enterprises play a fundamental role, with volunteers acting as the service network [10]. At the same time, such a platform should also reflect the social benefits of the enterprises and the public interests of all parties [11]. 

**Figure 1:**
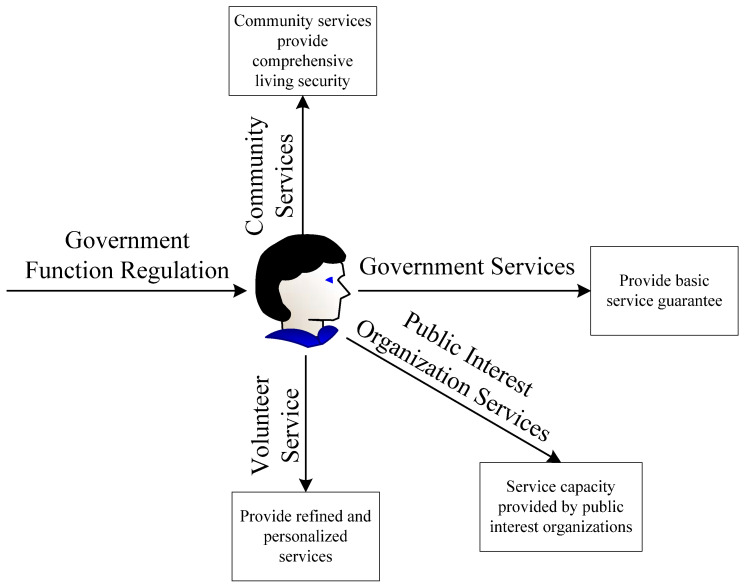
Platform service model diagram.

The sports public welfare platform aims to help people who were disabled due to participation in sports and those who are disabled but want to participate in sports and consists of five business chains, both internal and external, including the government, enterprises, communities, public welfare organizations, and volunteers. The five business chains are connected to each other and form a unified closed-loop market. The service targets are people who were disabled due to participation in sports and those who are disabled but want to participate in sports, for whom the government provides policies, funds, and other support, and the enterprises provide the resource pool for the China Disabled Persons’ Federation. As a grassroots unit, the community provides service guarantees, and non-profit organizations can provide comprehensive support services for the disabled. Volunteers dynamically and intelligently provide crowdsourcing services according to platform allocation [12]. Figure 1 shows the platform service model diagram. 

**Figure 2:**
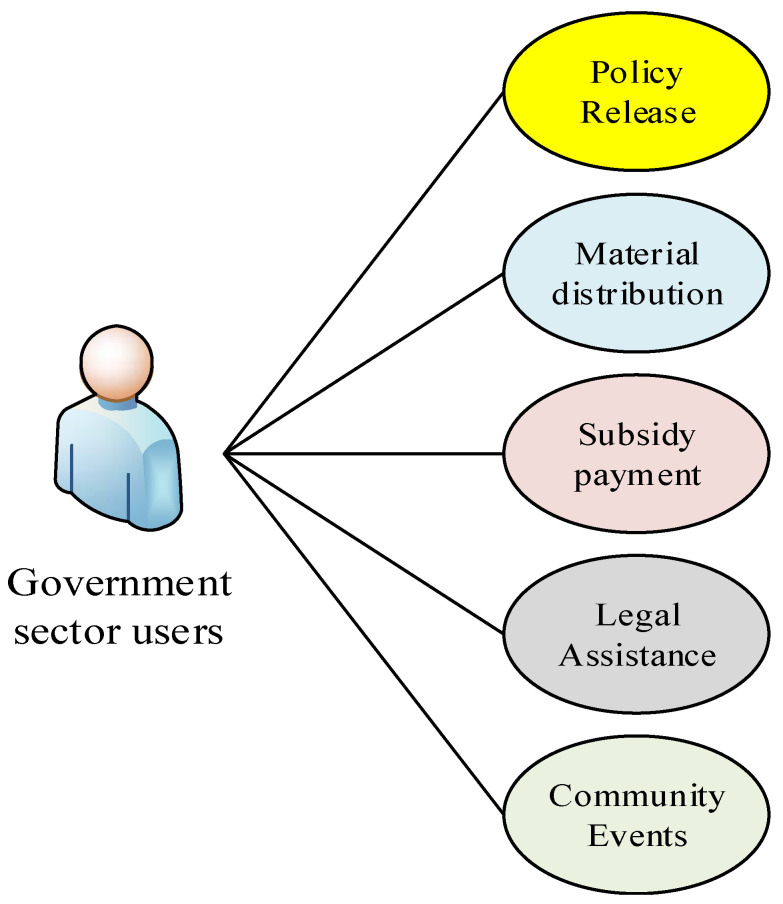
Diagram of government sector user requirements.

The government is in the central management position and attempts to meet various government sector user requirements, as shown in Figure 2. The use of any resources must conform with the rules set by the government, and the crowdsourcing model of volunteers also needs to be certified and reviewed by relevant government departments. The government is responsible for both offline support and online review. For offline support, the government provides funds for the disabled. The funds are allocated to the disabled resource pool through the disabled people’s federations, but it is not directly open to disabled people. This model avoids the improper distribution and waste of resources because of the unaudited use of funds [13]. Online review applies to all enterprises, volunteers, and disabled people who use the platform. One way this is achieved is via the use of different audit rules to audit. At the same time, all individual identities and behaviors are recorded, which allows for the formation of a big data warehouse overseen by the China Disabled Persons’ Federation, aids in the prediction of future trends, and enables the creation of next-step disability assistance plans [14]. 

In this platform, enterprises directly provide resources. The most important thing in assisting disabled people who want to participate in sports is the provision of various facilities and services. Disabled people often suffer from the inefficient use of funds and information silos, which may even result in wasting funds [15]. The offline and online rules for the disabled resource pool are determined in this platform. The resource pool is divided into two layers, the core layer and the surface layer. The former is the fund pool for helping the disabled. The latter is the disability assistance resource pool. The equivalent overlapping of the fund pool and the resource pool is used to build a complete disabled assistance service system. The realization of eliminating the overlapping use of resources relies on the various services provided by the enterprise, including facilities, equipment, labor, services, information, etc. All the above services constitute the outermost layer of the platform. The main structure supporting the system is the supply of the enterprises, which is called the peripheral ring [16]. The platform implements a controllable range of e-commerce systems with three built-in system management modules: the enterprise category, disability assistance service category, and enterprise access audit. Enterprises can apply for inclusion in the platform. During this process, they need to go through audits such as the selection of enterprise categories and the provision of service certificates. After auditing, they can enter the e-commerce management module of the platform. At the same time, the enterprises are divided into various categories and allocated service responsibilities. 

In the sports-related public welfare platform, which aims to help people who were disabled due to participation in sports and those who are disabled but want to participate in sports, community service carries out more functions of grassroots life management, coordination, and assistance, which is the last step in the implementation of the policy and a key link in guaranteeing services [17]. Strengthening and improving community service work is conducive to helping disabled people take part in various kinds of sports, improving their quality of life and promoting their all-around development. Social welfare organizations are non-governmental programs and do not prioritize profit maximization. They are the backbone of the disability assistance business chain. Integrating non-government organizations into the platform will provide strong and effective support for its users [18].

The most challenging part of providing sports-related public welfare activities for the disabled lies in the “last mile” of the service. If the service system is rooted in the commitment to passive obligations, there will be a lack of resource-driven services, making the market very inactive. Therefore, autonomous services are needed to change this status quo. We intend for this platform to use the volunteer network to solve the problem of the “last mile” [19]. From the perspective of social obligations, volunteers are the main body of autonomous services. If volunteers are provided with corresponding service compensation, including psychological and economic compensation, their enthusiasm will be greatly improved. Additionally, the crowdsourcing and crowdfunding system formed by the platform allows volunteers to independently choose their service direction and intensity according to their circumstances. Compensation is offered to volunteers based on their service direction and service intensity, which will form the basis for the marketization of services. Full use of market means can help realize the structuring and networking of services, thus changing the embarrassing status quo of assisting the disabled in the “last mile” [20].

The China Disabled Persons’ Federation and other government departments provide information databases of users’ information outside of the platform, including their identification and disability information. An interface is provided for the sports-related public welfare platform, and the government and the platform establish a link via the disabled fund account. After the account system is launched on the platform, it is responsible for confirming whether the subsidies provided to a disabled person are sufficient by considering their information and distributing the funds offered by the China Disabled Persons’ Federation to the disabled. It also has the function of annual clearing. 

The sports-related public welfare platform described in this paper offers a complete supply-and-demand business chain, with two layers of supply-and-demand relationships and two sets of connection paths. The value flow in the entire chain starts with government disability assistance funds or corporate sponsorship funds and ends with user consumption value. The service chain starts with users’ needs, makes proposals to the market under the control of the government through platforms and manufacturers, and returns to the original path. 

**Figure 3:**
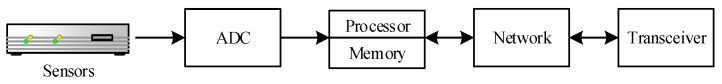
Sensor node architecture.

At present, due to objective factors, such as insufficient personnel and resources of the China Disabled Persons’ Federation and the requirement for refined service, multi-sensor technology is being introduced into the development of the sports-related public welfare platform. A single sensor node is limited in perception, processing, communication, etc., so sometimes multiple sensor nodes must be used simultaneously. The way the wireless sensor network processes information has changed, resulting in a new information processing mechanism: cooperative signal and information processing. By integrating the data of multiple sensor nodes, the network can perform operations that cannot be performed by a single node. Figure 3 shows the sensor node architecture.

Each node has independent sensing, processing, and communication functions, so each can perform its work independently. However, because of the limitations of a single sensor node mentioned above, the sensor network must cooperate with sensor nodes to improve its performance. In a wireless sensor network composed of thousands of nodes, it is impossible to directly control all sensor nodes. Under normal circumstances, a single node cannot obtain the entire network’s information. Therefore, in practice, the method of single-node autonomy and multi-node cooperation should be adopted so that users no longer need to pay attention to a specific node to achieve partial or overall control of the network. Through cooperation, the rows between nodes can be coordinated to ensure the consistency of each node, and compared with the central control, the robustness and performance of the network can be effectively improved. In the process of collaborative information processing, collaboration is an important aspect. Kalman filtering is a cooperative information processing technology widely used in wireless sensor networks. The following are the Kalman filter-related formulas. Since a Kalman filtering algorithm regards the estimated signal as the output of a random linear system under the action of white noise, and its input–output relationship is given by the state equation and output equation in the time domain, this filtering method is not only applicable to the filtering of stationary random processes, but also particularly applicable to the filtering of non-stationary or stationary Markov sequences or Gaussian Markov sequences, so its application scope is very extensive; Kalman filtering and tampering are two time-domain filtering methods which use state space to describe the system. The process noise and measurement noise of the system are not the objects that need to be filtered. Their statistical characteristics are exactly the information that needs to be used in the estimation process. It is unnecessary to know the first and second moments of the estimated quantity and the observed quantity at different times. 


(1) $$M\left(k+1\right)=\mathrm{IM}\left(k\right)+\mathrm{Jq}\left(k\right)$$

I—state transition matrix

(2) $$E\left[q\left(k\right)\right]=0$$

(3) $$E\left[q\left(k\right)q{\left(p\right)}^{T}\right]=W\left(k\right)\alpha_{\mathrm{kp}}$$

$E\left[q\left(k\right)\right]$—total energy conservation
J—process noise distribution matrix
$W\left(k\right)$—Gaussian noise vector

(4) $$Z\left(k+1\right)=\mathrm{GM}\left(k+1\right)+y\left(k+1\right)$$

$Z\left(k+1\right)$—the observation vector of the sensor

(5) $$\hat{M}\left(k+1|k\right)=I\hat{M}\left(k|k\right)$$

(6) $$\hat{M}\left(k+1|k\right)=I\hat{M}\left(k|k\right)+Jq\left(k\right)$$

k—time
$\hat{M}\left(k+1|k\right)$—prediction error

(7) $$L\left(k+1|k\right)=IL\left(k|k\right)I^{T}+JW\left(k\right)$$

$L\left(k+1|k\right)$—prediction covariance

(8) $$\hat{Z}\left(k+1|k\right)=G\hat{M}\left(k+1|k\right)$$

(9) $$\tilde{Z}\left(k+1|k\right)=Z\left(k+1\right)-\hat{Z}\left(k+1|k\right)$$

(10) $$D\left(k+1\right)=\mathrm{GL}\left(k+1|k\right)G^{T}+R\left(k+1\right)$$

$D\left(k+1\right)$—observed error covariance

(11) $$L_{mz}\left(k+1\right)=L_{mm}\left(k+1|k\right)G^{T}$$

(12) $$K\left(k+1\right)=L\left(k+1|k+1\right)G^{T}R^{-1}\left(k+1\right)$$

$K\left(k+1\right)$—filter gain type

(13) $$y\left(k\right)={\left[y_{1}^{T}\left(k\right),y_{2}^{T}\left(k\right),\cdots y_{B}^{T}\left(k\right)\right]}^{T}$$

v—noise

(14) $$Z\left(k\right)=G\left(k\right)m\left(k\right)+y\left(k\right)$$

$Z\left(k\right)$—single-cluster measurement model

(15) $$m_{u}\left(k+1\right)=m_{u}\left(k\right)+\mu \sum_{v\in B_{u}}i_{uv}\left(m_{v}\left(k\right)-m_{u}\left(k\right)\right)$$

(16) $$m\left(k+1\right)=\mathrm{Lm}\left(k\right)$$

μ—step size
L—non-negative matrix

Data fusion is also called information fusion or multi-sensor data fusion. It is difficult to give a unified and comprehensive definition of data fusion. With the development of data fusion and computer application technology, according to international research results, multi-sensor data fusion can be more accurately defined as follows. It makes full use of multi-sensor data resources in different times and spaces, and uses computer technology to analyze, synthesize, dominate, and use multi-sensor observation data obtained through time series under certain standards. It provides a consistent interpretation and description of the tested object to achieve corresponding decisions and estimates, so that the system can obtain more sufficient information than its components.

**Figure 4:**
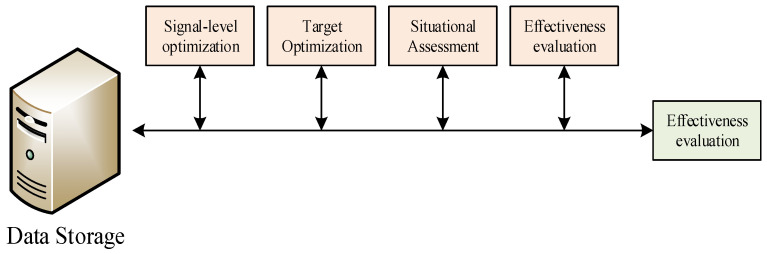
Data fusion model.

The multi-sensor data fusion system has better fault tolerance performance and increases the system’s reliability. An improvement in system detection can only reflect information about a particular aspect of the object by a single sensor. In a multi-sensor system, on the one hand, by using several identical sensors, the estimation of the target position and target velocity by a single sensor can be improved. On the other hand, by using a variety of sensors, the target object can be reflected from multiple aspects, which improves the reliability of the system detection and reduces uncertainty. The relatively poor reliability and accuracy of the information provided by a single sensor affect the evaluation of the surrounding environment and the decision-making process. A multi-sensor system can obtain more object information than a single sensor in the same amount of time. It not only improves the real-time performance of the system, but also reduces the cost of obtaining information and improves the system’s economy. As shown in Figure 4, a data fusion model is used to collect data related to sports for the disabled.

## 4. Practical Experiment of Sports-Related Public Welfare Platform

**Figure 5:**
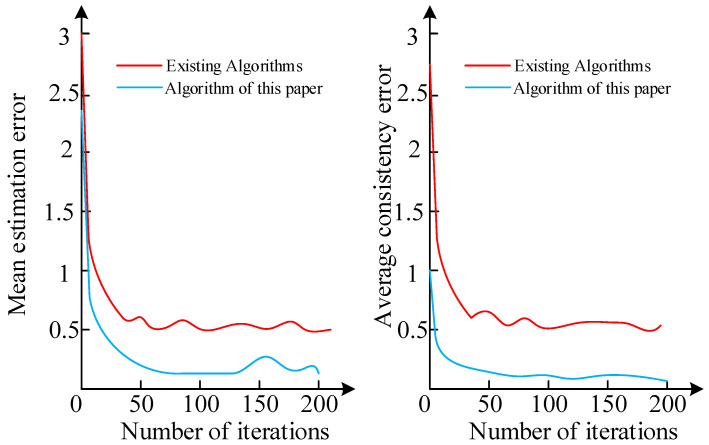
Average estimation error and average consistency error of two algorithms.

To verify that the multi-sensor data fusion algorithm proposed in this paper has better estimation performance, it was compared with the existing Kalman consistent filtering algorithm in terms of average estimation error and average consistency error. The simulation results are shown in Figure 5.

It can be seen from the figure that the average estimation error and average consistency error of the algorithm in this paper are both smaller than those of the existing algorithm. When the number of iterations is 0, the average estimation error of the existing algorithm is 3, and the average estimation error of the algorithm in this paper is 2.37.

**Figure 6:**
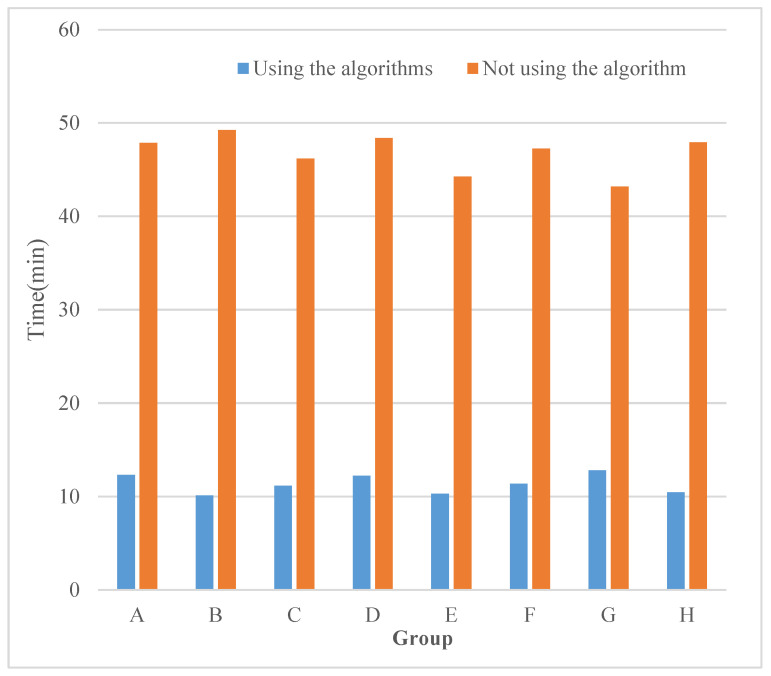
Rate experiment comparison results.

The multi-sensor data fusion algorithm proposed in this paper was used to collect and analyze the relevant data through the network, media, and other aspects. A comparison of the rate of data collection and analysis by the platform with and without using the multi-sensor technology detailed in this paper was made. Figure 6 shows the experimental comparison results. It can be seen that the time needed for collecting and analyzing data using the multi-sensor data fusion algorithm described in this paper was 12.3, 10.1, 11.15, 12.21, 10.29, 11.36, 12.81, and 10.46 min, which is significantly less compared to the time required when this algorithm was not used.

**Figure 7:**
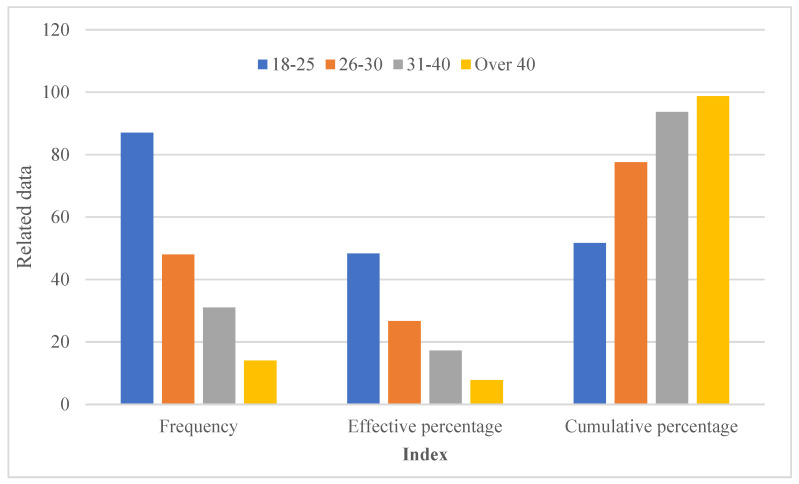
User age statistics.

As a platform that aims to help people who were disabled due to participation in sports and those who are disabled but want to participate in sports, how to help users of the platform to donate and ask for help more effectively and how to meet users’ needs for public welfare behavior are particularly important. Factors affecting users’ practical satisfaction with the sports-related public welfare platform were studied. Table 1 and Figure 7 show descriptive statistics of basic demographic characteristics.

It can be seen that in the sample data, young people aged 18–25 accounted for the largest proportion, accounting for 48.33% of the total sample, people aged 26–30 accounted for 26.67%, people aged 31–40 accounted for 17.22%, and people aged over 40 accounted for 7.78%.

Table 2 presents the usage of the user payment platforms. It can be seen that 71.67% of the respondents chose to use Alipay, and 23.89% of the users used Tenpay.

**Figure 8:**
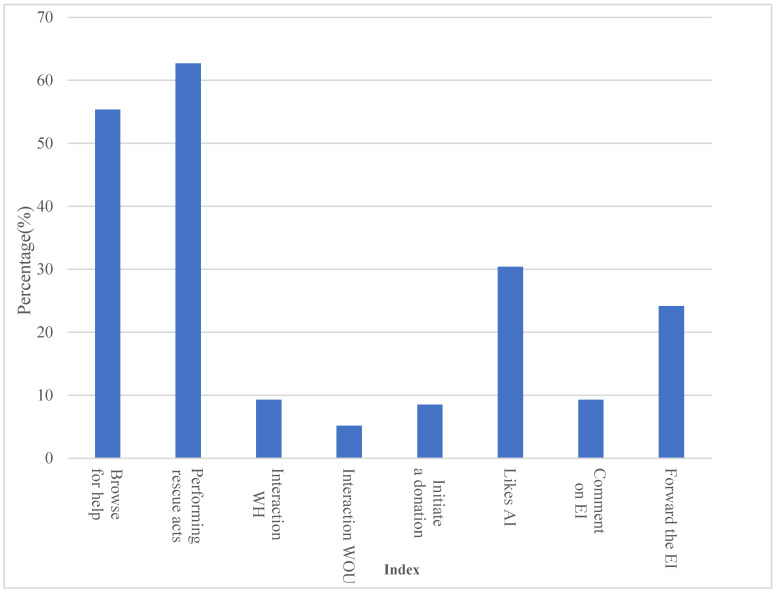
Statistical information on the use of the platform.

Regarding the activities users carried out on the sports-related public welfare platform, 62.67% of the subjects made public donations, 55.34% of the subjects browsed the donation information, 30.41% liked the public welfare information, and 24.16% forwarded the donation information. It can be seen that most users donated directly, but there were a few likes, reposts, and comments on the donation information posts, and most of them actively browsed the donation information. In addition, users of the sports-related public welfare platform rarely interacted with other users on the platform. Statistics are shown in Figure 8.

The satisfaction of users of the public welfare platform is shown in Table 3. The *p* value is the correlation coefficient. *p* < 0.005 indicates that the indicators are positively correlated. It can be seen that user satisfaction was above 0.75.

According to the above findings, the sports-related public welfare platform and other public welfare media platforms should pay attention to the aid effect. When users can really perceive the importance of their public welfare donations to the recipients and how their donations help others, their perception of social value increases, improving user satisfaction and promoting their donation behavior. Therefore, sports-related public welfare platforms need to provide timely feedback on users’ donations, follow up on the status of donation projects, and help users perceive the effectiveness of their assistance. At the same time, the platform could emphasize the achievement of users’ feeling of self-worth. When the user’s love is encouraged and commended, a sense of achievement of self-worth is randomly generated, which will also promote users’ public welfare donation behavior.

In terms of information quality, the sports-related public welfare platform and other public welfare media platforms should specially make efforts to ensure the reliability and timeliness of public welfare assistance information. Due to the rapid development of the Internet, information dissemination is accelerating. Fake information has flooded the Internet, making it hard to tell what is true or false. At the same time, fraudulent donations also occur frequently. Therefore, public welfare media platforms should identify the reliability of the information from the source, so as to avoid a crisis of trust among users. Public welfare media platforms should try their best to improve the disclosure and feedback mechanism of public welfare assistance information and donation use information, and more proactively disclose information to improve user supervision. This can increase user interaction and communication. A message for help on public welfare media platforms can be forwarded many times, and more users will leave messages and donation records when making donations. Users’ perception of mutual aid will be particularly obvious in such a charitable atmosphere.

Regarding system quality, sports-related public welfare platforms and other public welfare media platforms should ensure that users have convenient access to these platforms, the system is smooth, and user privacy and payment security are guaranteed.

## 5. Conclusions

With the Internet’s development, more and more platforms for public welfare activities have been made available. In platforms allowing people to make public donations, helping users donate and ask for help more effectively and meeting users’ needs for public welfare behaviors are particularly important. For this reason, this paper introduced multi-sensor technology and conducted research on sports-related public welfare platforms. This paper attempted to integrate the forces of the whole society, including the disabled, volunteers, government departments, enterprises, and social institutions, to develop a sports-related public welfare platform to help people who were disabled due to participation in sports and those who are disabled but want to participate in sports and help improve user satisfaction with the platform. The experimental results show that the multi-sensor data fusion algorithm dramatically improves the efficiency of data collection, and the sports-related public welfare platform designed in this paper has good user satisfaction. Given the limited data sources and academic level, there are inevitably omissions in the research. The analysis of the status quo was not thorough enough. It only shows the changes in relevant indicators and lacks internal judgment analysis. In the theoretical research stage, a more comprehensive understanding of the theory is needed. Whether the model can be applied to small cities below the third tier should be verified through further research.

## Figures and Tables

**Table 1:** User gender statistics.

	Frequency	Effective Percentage	Cumulative Percentage
Male	38	22.35	22.35
Female	132	77.65	77.65
Total	180	100	100

**Table 2:** Usage of the user payment platforms.

	Frequency	Effective Percentage	Cumulative Percentage
Alipay	129	71.67	72.5
Paypal	43	23.89	95.4
Wing Pay	4	2.22	96.1
Others	4	2.22	99.4

**Table 3:** Path factor.

	Satisfaction	*p*
Satisfaction (Services)↔User expectations	0.795	0.001
Satisfaction (Information)↔User expectations	0.864	0.001
Satisfaction (Social)↔User expectations	0.852	0.002

## Data Availability

Data will be made available on request.
